# A Case of Supposed Hydrophobia

**Published:** 1875-02-01

**Authors:** B. M. Wilson, A. C. Corr

**Affiliations:** Sec’y.; Sec’y pro tem.


### A CASE OF SUPPOSED HYDROPHOBIA.

*From the Carlinville, Ill., Democrat.*

THE following article relating to the recent case of *supposed hydrophobia* in this city, was read before the County Medical Association at Shipman on Tuesday, by Dr. J. P. Matthews, of this city, and will no doubt interest many of our readers:

Not so much on account of any peculiar friendship for the poor canine race does this report appear, as a duty incumbent upon every physician to point out to the human family all forms of physiological and hygienic errors wherever or whenever they may occur. This is especially true, when observed by a practitioner of medicine to occur in the families with whom he stands in the relation of family physician. My professional friends will probably feel sufficiently interested to give the views expressed a brief attention.

A case simulating and in some respects resembling hydrophobia has recently occurred in our city, which many, unacquainted with the pathology of the disease, believe to have been a true case of the dreaded malady. The case in the son of Mr. Shay, came first under the observation of the writer as his family physician soon after the inception of what were peculiar nervous phenomena, but a careful inquiry as to the dog that had bitten the boy six weeks previous, revealed the fact that he was not only then, but had been in excellent health. This, together with the fact that the boy could not only drink water, but was relieved from the paroxysms by the application of cold water to his head, precluded in the writer's mind the possibility of its being a case of hydrophobia. For this opinion expressed to the friends, the writer was discharged and others attended, whose diagnosis and prognosis were more in unison with the boy's surroundings.

The opinion, that it was a species of delusional insanity, supervening upon the biting of a healthy dog, six weeks previous, which again attacked but did not bite him, four weeks subsequent to the biting, all occurring too, upon the arrest of a chronic discharge from one ear, the latter giving rise to an irritation of the brain and nervous system, has been undoubtedly confirmed by the sequel of the case. A careful report which embodied the above opinion, was sent by the writer to Prof. William A. Hammond, of New York, than whom, no man living to-day, stands higher upon diseases of the mind and nervous system. His opinion, which was requested, is herewith appended:

Jan. 10th, 1875.

*Dear Doctor:* The case you describe is undoubtedly one of hysterical or imaginary hydrophobia, many cases of which occur here every year.

There is not a single element of real hydrophobia about it.

Your view of the case does not differ in any respect to mine.

Yours, Respectfully,
WILLIAM A. HAMMOND.

The report was submitted to Prof. Hammond because he has, in the last year, in his careful and profound investigations of the nervous system, made the disease under consideration a special study, and it must not be forgotten, that he in his report to the New York Neurological Society, was the first to point out an actual lesion in the root of the spinal chord (medulla oblongata) produced in the cases of death caused by hydrophobia.

The secondary cause, then, or the cause of the pathological phenomena of the disease has been discovered by scientific research; but the peculiar principle or virus—the inoculation into the circulation, of which produces it, science as yet fails to point out. This principle or poison that exists upon the teeth of the rabid animal, acts as specifically upon the medulla oblongata as that of small-pox upon the skin, diphtheria upon the throat or cholera upon the intestinal canal, and when that virus is not present a symptom resembling it, is no more hydrophobia than tetter or acne is small-pox, a sore throat from cold is diphtheria, or a mere diarrhœa is cholera. A conclusion in the above case was reached from this course of reasoning, and was regarded sufficient without the symptoms as they appeared, inasmuch as the boy was bitten by a perfectly healthy dog.

The authors upon the subject, at the writer's command, are of no less note than Watson, Aitkin, Flint, Williams, Hartshorne, etc.; all give the particular symptoms to be "severe constriction about the throat; spasmodic action of the diaphragm and a particular distress at the stomach, all of which are aggravated or brought about by attempts to take liquids, or by the least breath or current of air on the surface of the body, which produces in the first instance an effect resembling that produced upon stepping into a cold bath. Tenacious and clammy mucus issues from the mouth, paroxysms of frenzy or uncontrollable, impulsive violence (rabidity) supervene. The duration of the disease varies from three to six or seven days, the greater number of cases terminating in death on the second and fourth days from the accession of the symptoms. Death is generally sudden and unexpected at the moment."

The authors also agree that the first stage of hydrophobia is ushered in by the patient's attention being aroused by a numbness extending from the base of the brain to the wound, which, if it occurs in the hand or foot, often produces a tremulous feeling, slight fever and headache, which last but a short time before the hydrophobic stage begins.

In the case under consideration, restlessness at night and dreaming of dog constituted the nervous symptoms that occurred before the paroxysms, and even with a pain in the side of the forehead, which has been accounted for in the arrest of the purulent discharge from the ear, the writer could not at this time see any reason for forming so grave a diagnosis.

The authors also agree that the second or hydrophobic stage is ushered in as the word implies, with a great difficulty, if not an utter impossibility of swallowing liquids, a symptom also not present in Mr. Shay's boy. In fact the only real symptom of the malady that occurred was the snapping and frothing at the end of the paroxysms which the writer has seen, and do occur in prolonged epileptic fits.

This, then, could not have been a case of hydrophobia; inasmuch as science teaches us that it is a result of a specific poison introduced into the circulation.

Dr. Condie, in a note in his edition of Watson's Practice, says that many well authenticated cases are on record in which disease having all the pathognomonic symptoms of hydrophobia occurred without the slightest evidence of having been bitten by a rabid animal, but this was written years before the above referred to investigations were made upon the subject; and Youatt, in a pamphlet upon hydrophobia, believes it to be communicable only by the dog, the fox, the wolf, jackal and cat.

A bite from a healthy dog then, is no more to be feared than any other contused wound; but if the dog is suspected with having the disease, cauterize the wound freely with caustic potash, or lunar caustic, or what would be still safer, immediately excise the part with a knife. This is recommended as the safest preventive. As to curative agencies, the writer is sorry that science as yet offers but little relief. This fact makes the cases fit pabulum for snapping doctors, magic and mad-stones, the latter of which is given as an ancient tradition by Dr. Charles P. Russell, in an article on some superstitions on hydrophobia in the December number of the *Popular Science Monthly*. He refers in his article to the superstition relative to dog-days, the cutting a worm from beneath the dog's tongue, and biting off the dog's tail, both operations to be performed in puppyhood, to prevent the mature dog from going mad.

He says "The ancients ascribe peculiar virtues to a variety of stone called *ammonis cornu*, which was supposed to possess the property of extracting the virus from wounds inflicted by mad dogs or venomous reptiles. Pliny alludes to it under the above name, and it has since received the appellation ammonite, both terms referring to its resemblance in shape to the horns which surrounded the head of Jupiter Ammon. It has also in more modern times been popularly known as the mad-stone and the snake-stone. Scientifically speaking, it is the fossil petrifaction of an extinct mollusk, closely resembling nautilus, having a spiral, symmetrical and chambered shell, ranging in size from that of a small bean to that of a large cart-wheel. In the East Indies and China, it has for ages enjoyed the reputation mentioned."

And after speaking, as a hocus-pocus, of a Chinese snake-stone that was brought to this country about A. D. 1740, he says: "These wonderful stones doubtless still exist with virtues unimpaired—a profitable inheritance for those whose privilege it is to bestow their inestimable boon upon credulous humanity."

Dr. Seaman.—I saw the Shay case referred to in the report, and also the so-called mad-stone. It is a hornblende prepared stone. Does not appear porous; of a bluish color and striated. It did not stick sufficiently to prevent it falling by its own weight. The hand was the part it was applied to, and when the hand was allowed to turn to one side the stone would roll off. Its sticking is purely mechanical. I endorse the views embodied in the report.

I had a case like the one referred to, occurring in a fitty family, which was supposed by the friends to be hydrophobia, but was hysteria. The case lived, but had many attacks.

Dr. Wm. C. Day.—I saw a mad-stone which was exhibited to the medical class in St. Louis, the keeper of it being present. It was white, about half as large as a turkey egg, very porous, finely polished, chalky appearance. It was kept in wax. Prof. J. T. Hodgen made it the subject of a lecture. Said it was one of the humbugs of medicine; that its sticking is purely mechanical, depending on adhesion and capillary attraction, and not on any affinity for the poison.

Dr. Harris.—I have seen the stone referred to by Dr. Seaman. It is essentially as he described it.

Dr. A. B. Penniman.—Gave a verbal report of a case of unusual nervous trouble occurring in a person who had been bitten by a dog, several years before. The attacks, which were called "mad-fits" by the friends, occurred about three times annually, at irregular intervals. I did not believe it to be hydrophobia.

Dr. Matthews.—It will be remembered the stone referred to by Dr. Seaman, was applied in a case of *true* hydrophobia, in Carlinville, a few years ago. The patient was a German, whose name I have forgotten, but was a patient of Dr. DeLieu's. The man died in a few days.

Other opinions were expressed, which were about as follows:

That there are cases of hydrophobia there is no doubt, but they occur much less frequently than people suppose. The mad-stone is a traditional superstition, like dog-days, the signs of the Zodiac as controlling the circulation of the blood, etc., having no facts in science to support them.

The meeting of the Society was well attended. Many subjects of importance were discussed; most conspicuous was an essay on Cerebro-Spinal Fever.

Society adjourned to meet in Carlinville, third Tuesday in April next.

B. M. WILSON, Sec'y.

A. C. CORR, Sec'y *pro tem.*

